# Exploratory Reanalysis of the Lithuanian OHIP-14 in Older Adults: Preliminary Evidence for an Oral Discomfort Structure

**DOI:** 10.3390/medicina62101844

**Published:** 2026-09-23

**Authors:** Yvonne Buunk-Werkhoven, Mantė Kireilytė, Daiva Mačiulienė, Gediminas Žekonis, Žana Sakalauskienė

**Affiliations:** 1 Faculty of Health Sciences, Oral Health Center, Kauno Kolegija Higher Education Institution, K. Petrausko st. 15, LT-44162 Kaunas, Lithuania; 2 Faculty of Prosthodontics, Lithuanian University of Health Sciences, Sukileliu pr. 51, LT-50106 Kaunas, Lithuania; kireilyte.mante@gmail.com (M.K.); gediminas.zekonis@lsmu.lt (G.Ž.);

**Keywords:** OHIP-14, psychometrics, reanalysis, Lithuanian older adults, oral discomfort, psychological discomfort, physical discomfort

## Abstract

*Background and Objectives*: This study is a secondary analysis of a Lithuanian version of the original Oral Health Impact Profile-14 (OHIP-14) in a sample of older adults in Lithuania and, through factor analysis, it derives one or two practical and easily interpretable measurement instruments for use in clinical practice and applied research. *Materials and Methods*: The dimensional structure and reliability of the subscales resulting from this reanalysis, conducted among 54 respondents with a mean age of 68 years (*SD* = 10.2, range 49–90), were examined. *Results*: Exploratory factor analysis (EFA) with Varimax rotation of the Lithuanian OHIP-14 scale showed a three-factor solution; however, the fixed two-factor solution, consisting of psychological discomfort (6 items) and physical discomfort (5 items), tested with EFA with Varimax rotation was satisfactory, and the one-factor solution, oral discomfort (comprising 11 items), was also acceptable. Age was the only sociodemographic factor significantly associated with oral discomfort. Participants younger than 69 years reported higher levels of overall oral discomfort, psychological discomfort, and physical discomfort compared with those aged 69 years and older (all *p* ≤ 0.002). No significant associations were observed for denture status, gender, education, income, or marital status. *Conclusions*: After modification and adaptation to the target group, the Lithuanian OHIP-14 may have potential as a measure of oral discomfort, with preliminary findings suggesting a distinction between psychological and physical dimensions. These findings should be considered exploratory, given the limited sample size, and provide a basis for the further translation and adaptation of the existing Dutch Oral Discomfort Scale, but they require confirmation in larger studies before the proposed structure and broader applicability of the instrument can be established.

## 1. Introduction

A 2025 scoping review concluded that comparisons of findings across studies that use the Oral Health Impact Profile (OHIP) require careful consideration of differences in instrument design and content [1], as variations between OHIP versions may influence measured outcomes. Among the eleven generic OHIP scales developed for broad application, rather than targeting specific conditions, a two-dimensional scale [2] represents the most recent addition, reflecting ongoing efforts to refine the assessment of oral-health-related quality of life (OH-QoL) [3]. OH-QoL is a broad, multidimensional concept describing how oral health can affect an individual’s physical, psychological, and social functioning and well-being. In 2018, a factor analysis of the Dutch version of the OHIP-14 conducted in a small and vulnerable psychiatric patient population identified three distinct dimensions reflecting functional, social, and psychological aspects of oral health-related quality of life [4]. In 2025, using a broad and diverse sample, Buunk-Werkhoven et al. examined a single construct to determine the degree of an individual’s own perceived oral discomfort (OD), consisting of two related subscales, psychological and physical [2]. OD, by contrast to OH-QoL, refers more specifically to an individual’s subjective experience of unpleasant, bothersome, or distressing oral complaints and their physical and psychological impact, and can thus be viewed as a more specific and concrete concept within the broader domain of OH-QoL. Since the original OHIP-14 [5] can be considered as a domain sample of the broader OH-QoL construct, the use of a single overall sum core, combining all 14 items into a unidimensional measure despite their representation of seven dimensions, may not fully reflect the multidimensional nature of self-reported quality of life related to oral health problems [1,2,3,4,5]. Therefore, interpretation of the total OHIP-14 score as a single indicator of OH-QoL should be made with caution, particularly when the underlying dimensional structure is not adequately supported.

At the same time, the growing number of available OHIP versions highlights the need for caution when developing new adaptations. While expanding the instrument may seem beneficial, unnecessarily proliferation can hinder standardization and comparability across studies. Selecting the most appropriate existing version is therefore a crucial step for researchers. This decision becomes more important given the significant time, cost, and methodological accuracy required to adapt and validate an OHIP instrument use in different languages and cultural contexts. Additionally, shorter and more targeted questionnaires may be beneficial in clinical practice by reducing respondent burden and completion time while facilitating a more focused assessment of patient-reported outcomes [1,3,5,6].

In line with the reasons mentioned above, the Lithuanian OHIP-14 version [7] used also has a similar number of limitations from a psychometric point of view [2,3,4]. First, because the pilot study focused primarily on feasibility and comprehensibility, consequently, not all OHIP-14 dimensions were represented in the Lithuanian pilot sample. However, the full psychometric properties of all dimensions were subsequently evaluated and reported in the main pilot study [7]. Second, although a Cronbach’s alpha of 0.924 was considered excellent, its use for a two-item scale is debatable [1,2,3], given that alpha largely reflects the correlation between the items. The order of the questions can influence scores, and violated assumptions may lead to suboptimal reliability, especially for multidimensional constructs [8].

As previously suggested, the OHIP-14 may be more appropriately interpreted as reflecting OD [2] rather than a comprehensive measure of OH-QoL. The broader literature [1,3] also points to this psychometric issue; our recent OD scale offers a potential conceptual answer to this. Therefore, the present secondary analysis examines whether the Lithuanian OHIP-14 can serve as a suitable basis for deriving dimensions related to OD among older adults in Lithuania [7]. This analysis represents a necessary preparatory step for the subsequent translation and adaptation of the existing Dutch Oral Discomfort Scale [2,9] to the Lithuanian context among older adults in Lithuania [7].

## 2. Materials and Methods

### 2.1. Ethics and Consent to Participate

As in the published pilot study, ethical approval and permission were granted by the Centre for Bioethics at the Lithuanian University of Health Sciences (LUHS) (No. BECOF-538; 26 February 2015). This secondary study with human participants, who had been informed of the purpose of the original study and from whom informed consent had been obtained, was also conducted in accordance with the universal ethical principles of the Declaration of Helsinki.

### 2.2. Study Sample

The dataset used of the Lithuanian sample included 54 participants (68.5%, *N* = 37 female; 31.5%, *N* = 17 male) who received dental treatment at the Department of Prosthodontics (LUHS, Kaunas) in March 2015 [7]. In the current exploratory study, the two 49-year-old respondents who were excluded from the previous study have been included. Their mean age was 68 years (*SD* = 10.2, range 49–90); notably, no participants were aged 68 or 69 years. Information was collected on denture status, age, gender, income, educational level, and marital status. Since age was assessed as a continuous variable and denture status as a dichotomous variable (yes/no), participants in this secondary study were categorized by denture status (yes/no) and age (≤68 vs. ≥69 years). This age cut-off point was selected based on the sample mean, aligning with the clinical study using the Dutch OD scale [9], rather than on a pre-established clinical or population-based threshold.

### 2.3. Measures

#### Short Version of the Lithuanian OHIP

To re-assess the scale, all items of the Lithuanian version of the OHIP-14 were used. This translated instrument consists of 14 items organized into seven two-item dimensions, with the original answer options [1,5]. The response options of the original Lithuanian version of the OHIP-14 were recoded, in line with the answer option of the Dutch version of the OHIP-14, from which the Dutch OD scale is derived [2,4].

For this secondary study, a general one-dimensional score was calculated as the sum of the 11 items used in the Dutch OD scale [2]. In addition, sum scores were computed for the two-dimensional scale, comprising *psychological discomfort* and *physical discomfort* for each respondent. Regarding the calculation of sum scores, respondents with missing data for one or more items were excluded from the calculation of these total (sub)scale scores, in accordance with psychometric principles. Removing items from the OHIP scale may affect reliability estimates and is justified only when a clear rationale exists and face validity is maintained [8]. Therefore, Guttman’s lambda 2 coefficient was also used here as an alternative measure of reliability, alongside Cronbach’s alpha. The number of valid responses varied across (demographic) variables due to missing participant information and/or because not all participants had answered every question in the questionnaire. Consequently, the number of valid responses varied by individual item and by analysis. Missing responses were not imputed and were excluded from the relevant analyses.

### 2.4. Statistical Analyses

The used data collection was provided by Kireilyte et al. [7] and downloaded from Excel into an IBM Statistical Package for Social Sciences 29.0 (SPSS Inc., Chicago, IL, USA) file for data reanalysis. Unlike the original study, which used ANOVA and chi-square tests, this reanalysis applied exploratory factor analysis (EFA) to examine the latent structure of the instrument. The Kaiser–Meyer–Olkin (KMO) measure of sampling adequacy was 0.83, indicating good suitability of the data for this factor analysis. Bartlett’s test of sphericity was significant, χ^2^(91) = 442.53, *p* < 0.001, indicating that the correlations among the items were sufficient for this factor analysis. The internal consistency of the derived (sub)scales was assessed by Cronbach’s alpha (α) and lambda 2, and *t*-tests were performed to determine whether there were any significant group differences. The cut-off level for statistical significance was set at *p* < 0.05.

## 3. Results

### 3.1. Reanalysis of the Lithuanian OHIP-14

First, the structure of the recoded Lithuanian OHIP-14 was re-examined, and respondents (*N* = 8) with a missing value for any of the items were excluded from the scale total score in the analyses. The mean, standard deviation, and range of the total score on the recoded short Lithuanian OHIP version (*N* = 46, 8 missing) were computed (M = 19.39, *SD* = 14.32, range 0–49), and the distribution of scores was skewed distribution to approximately normal.

### 3.2. Exploratory Factor Analyses

Based on an exploratory factor analysis with Varimax rotation of all 14 Lithuanian OHIP-items, a founded three-factor solution explained 70.30% of the variance. Three original OHIP-items [1,5,7], i.e., Q1, “Have you had trouble pronouncing any words because of problems with your teeth, mouth or dentures?”, Q2, ‘Have you felt that your sense of taste has worsened because of problems with your teeth, mouth, or dentures?’, and Q14, “Have you been totally unable to function because of problems with your teeth, mouth, or dentures?”, were excluded based on conceptual misalignment with the Dutch oral discomfort construct [2]. The remaining 11 Lithuanian OHIP items are described in Table 1.

A two-factor solution as described in previous studies [2,9] consisting of psychological oral discomfort (six items from the short Lithuanian OHIP version; Q5, Q6, Q9, Q10, Q11, and Q13) and physical oral discomfort (five items from the Lithuanian OHIP-14 version; Q3, Q4, Q7, Q8, and Q12) was found to be satisfactory. The first factor had an eigenvalue of 6.49 and explained 59.0% of the variance, while the second factor had an eigenvalue of 1.02 and explained 9.2%. Together, the two factors accounted for 68.2% of the total variance. Item communalities ranged from 0.53 to 0.83, indicating that the retained factors adequately explained the variance in the individual items. Ten out of eleven items loaded above 0.55 on one of the two factors and did not load substantially on the other factor. Although the KMO was good, given that the sample size in this reanalysis is relatively small (the ratio of participants to items is approximately 54/14 = 3.9) and is further limited by cases with missing data, the factor-analytic results should be viewed as exploratory or preliminary. Therefore, the structure of the two-factor model identified in the current study was not entirely consistent with previous Dutch findings [4]. Some items appeared to represent more than one aspect of oral discomfort. For example, the item “Have you been self-conscious because of your teeth, mouth, or dentures?” showed high loadings on both factors. In addition, two items, “Have you had difficulty doing your usual jobs because of problems with your teeth, mouth, or dentures?” and “Have you had to interrupt meals because of problems with your teeth, mouth, or dentures?”, loaded more strongly on the psychological oral discomfort factor, although these experiences may also reflect physical limitations. These items are multi-interpretable and may depend on the individual’s personal experience, the cultural context, and the wording of the translated items.

### 3.3. Internal Consistency and Reliability Analysis

Internal reliability was examined using item–total correlations and Cronbach’s alpha if an item was deleted (Table 1). For the subscale psychological discomfort (six items), Cronbach’s alpha was 0.93 and Guttman’s lambda 2 was 0.94. For the subscale physical discomfort (five items), Cronbach’s alpha was 0.76 and Guttman’s lambda 2 was 0.79. The one-factor solution, oral discomfort (comprising the aforementioned 11 items, excluding Q1, Q2, and Q14 in the original Lithuanian OHIP-14 version) demonstrated excellent reliability, with a Cronbach’s alpha of 0.93 and a Guttman’s lambda 2 of 0.94.

The OD scale (11 items) and its subscales *psychological discomfort* and *physical discomfort* consisting of 6 and 5 items, respectively, were found to be not only statistically but also theoretically adequate.

The mean sum scores were calculated for the one-dimensional scale and for each subscale with higher scores indicating greater perceived OD (*N* = 47; 7 missing; M = 16.66; *SD* = 12.03; range 0–38), *psychological discomfort* (*N* = 51; 3 missing; M = 9.41; *SD* = 7.39; range 0–24), and *physical discomfort* (*N* = 49; 5 missing; M = 7.12; *SD* = 4.94; range 0–16).

### 3.4. Group Comparisons

Correlational analyses were carried out to establish the direction and magnitude of the associations between the sociodemographic variables—denture status, age, gender, marital status, education, and income—and the main outcome variables. Significant differences were observed only between the two age groups: participants younger than 69 years reported higher levels of OD (*N* = 21; M = 23.29; *SD* = 11.60; 95% CI [18.01, 28.57]) than those aged 69 years and older (*N* = 26; M = 11.31; *SD* = 9.59; 95% CI [7.44, 15.18]); t(45) = 3.88; *p* < 0.001. The younger group reported higher levels of oral discomfort than the older group (Cohen’s *d* = 1.14; Hedges’ *g* = 1.12). Moreover, the younger age group (*N* = 24) reported significantly higher *psychological discomfort* (M = 13.13; *SD* = 7.17; 95% CI [10.10, 16.16]) than the older age group (*N* = 27; M = 6.11; *SD* = 5.96; 95% CI [3.75, 8.47]); t(49) = 3.81; *p* < 0.001; Cohen’s *d* = 1.07; Hedges’ *g* = 1.06. The mean difference was 7.02 (95% CI [3.23, 10.81]), indicating a large effect that was observed across all six items of the *psychological discomfort* subscale.

In addition, *physical discomfort* was also significantly higher among younger participants (*N* = 23; M = 9.39 *SD* = 4.78; 95% CI [7.32, 11.46]) compared with participants aged 69 years and older (*N* = 26; M = 5.11; *SD* = 4.17; 95% CI [3.43, 6.79]); t(47) = 3.34; *p* = <0.001; Cohen’s *d* = 0.96; Hedges’ *g* = 0.95. The mean difference was 4.28 (95% CI [1.71, 6.85]), also indicating a large effect. No other significant effects were found of denture status, gender, education, income, or marital status on OD.

## 4. Discussion

In the present study, the dimensional structure and the reliability of a Lithuanian version of the OHIP-14 used in a pilot study [7] were re-examined, with the aim of deriving one or two scales to measure OD [1,2,9], which are content-valid, structured and easy to apply in practice. Following the aforementioned study by Buunk-Werkhoven et al. (2025) on the two-dimensional scale for OD [2], this secondary study focused on an exploratory factor analysis (EFA) with Varimax rotation of the structure of the recoded Lithuanian OHIP-14 version, which had previously been tested for reliability and validity using only the small pilot study sample [7]. The psychometric property analysis of the Lithuanian OHIP-14 instrument provided preliminary exploratory evidence consistent with a possible two-dimensional structure, with the dimensions *psychological discomfort* and *physical discomfort* appearing to fit the data. It should be noted that a six-month ‘recall’ period was used for this Lithuanian OHIP-version-derived OD scale, in which respondents were asked about their feelings and/or problems over the past six months, whereas no such a ‘recall’ period was specified for the oral discomfort scales used in earlier Dutch studies [2,9]. Recently, a scoping review of the OHIP as an instrument for assessing OH-QoL [1] reported that the measurement is sensitive to changes in item order and the method of administration. In the original OD scale [2,9], the Dutch version of the short OHIP has also undergone modifications in response options in 2009, namely, ‘sometimes’ and ‘regularly’ compared with the usual ‘almost never’ and ‘occasionally’ [1], while a study with the OHIP-14-NL [10] provides an earlier description of the Dutch instrument. This illustrates the importance of maintaining consistency in instrument administration and wording when comparing findings across studies and populations. In Lithuania in 2022, due to the growing interest in multidisciplinary treatment of patients with advanced stages (stage III–IV) of periodontitis, a 49-item OHIP questionnaire (OHIP-Lt) was translated [11]. In general, a longer questionnaire is more time-consuming to administer; for example, the OHIP-49 is estimated to take approximately 17 min to complete, which may limit its practical use [1,3]. Thus, for both clinical and research purposes, it is more adequate and feasible to use a two-dimensional scale, consisting of 11 items in total [2,9,12].

This secondary research has several limitations. A primary limitation of the current study was that the data were derived from relatively small and specific population of older adults. For instance, the number of valid responses varied depending on demographic variables, and consequently, the sample size for specific demographic analyses differed by variable. Historically, most published sample size recommendations [13] have been based on rules derived from expert experience; e.g., Gorsuch [14] and Kline [15] proposed absolute minimum numbers of at least 100 participants in their widely cited guidelines. Comrey and Lee (1992) classified sample sizes as follows: 50 = very poor; 100 = poor; 200 = fair; 300 = good; 500 = very good; and 1000+ = excellent [16]. Although sample sizes of 55–100 respondents are sometimes considered “acceptable,” their adequacy depends on the research context and study type (e.g., field studies or applied research) [4,9,17,18]. Nevertheless, for a thorough factor analysis involving 11 items, it is generally recommended to include at least 100–150 respondents to obtain reliable results [13].

Thus, the sample size of 54 used is considered very poor, meaning that the derived scales should be administered in the future to samples that are representative of the target population. This is also necessary to obtain normative values, which would allow users to assess the extent to which an individual, patient’s or group’s score is relatively high or low. Furthermore, limited reporting of discrepant N-values, missing data, and the processing methods employed complicate the assessment of the risk of bias and of the robustness, validity, and reproducibility of the reported results [19]; overall, this may have reduced the statistical power of some comparisons and potentially limited the generalizability of the findings. In particular, the finding that younger participants reported higher levels of oral discomfort than older participants warrants further consideration. A possible explanation may be that younger individuals may have different expectations, perceptions, or coping mechanisms regarding oral discomfort, which could influence their self-reported symptoms. However, given the exploratory nature of this study and the absence of a clinically established age limit (it was in line with [7]), this finding should be interpreted with caution. Confirmation in future studies with larger sample sizes and where age is considered a continuous variable is necessary.

A second limitation is that no data are currently available in Lithuania regarding the validity of these scales, which could be assessed, for example, by examining their relationships with similar instruments. The present findings make a first contribution to the ongoing discussion regarding how OH-QoL and OD are conceptualized and measured. By re-examining the structure of the Lithuanian OHIP-14, this study suggests that the instrument may capture distinct psychological and physical aspects of OD. This perspective may be relevant for future research, as the OHIP-14 is typically interpreted as a measure of the broader impact of oral health on quality of life [1,3], whereas the current findings suggest the possibility that some of the information captured by the instrument can be interpreted more specifically in terms of subjective OD [9,20].

A third potential limitation is that the practical usefulness of these scales has not yet been demonstrated among older adults living independently or in nursing homes. Quantitative research on the outcomes of oral disorders, as perceived and reported by patients, is required to further advance dental and oral care in Lithuania. Therefore, a Lithuanian-applicable OD measure should be adopted to ensure consistency in assessing and measuring outcomes that more explicitly address the issues and discomfort related to the mouth [2,9,11].

In practice, a Dutch version of the OD scale [2,9] translated into Lithuanian, carefully adapted to the characteristics of the target population, could provide a basis for assessing oral discomfort and related oral-health behaviors in the Lithuanian population [2,9,21]. Such assessment could subsequently inform interventions targeting oral well-being and related behaviors. Importantly, this does not imply a simple re-translation of existing instruments, but rather the application of a rigorous translation and intercultural adaptation procedure for this two-dimensional scale [22]. Following translation and intercultural adaptation, the Lithuanian version should undergo pilot testing and psychometric evaluation in the target population, including assessment of the psychological and physical subscales [10,22,23].

Applied research based on composite questionnaires, including measurements as indices and scales, is important in medicine [19], public (oral) health, education, and psychology because it allows efficient, standardized, and anonymous data collection from large populations. The OD scale supports a systematic assessment of subjective oral-health experiences, which contributes to personalized treatment planning and guides evidence-based public health policies aimed at preventing oral diseases and improving oral health among elderly and vulnerable populations [4,21,24].

The Problem–Analysis–Test–Help–Success model [25] provides a conceptual structured framework for translating oral-health research into tailored interventions [9,21], from identifying a problem or determinants to developing interventions and evaluating solutions. In the Problem phase, validated instruments such as the OD scale identify target populations and urgent needs, including older adults, nursing home residents, and other vulnerable groups [4,17,18]. The Analysis and Test phases examine determinants of oral discomfort and their relationships with healthcare systems, behaviors, and environmental factors [1,2,3]. These findings provide a basis for developing tailored oral-health education and promotion strategies within the Help phase [25,26,27,28]. The Success phase then considers the effectiveness of such interventions and their potential for broader application, including the promotion of oral self-care awareness and sustained behavior change [25,26]. Translated, validated, and culturally adapted instruments enhance comparability, international collaboration, and preventive outcome monitoring, while strengthening research credibility, reproducibility, ethical standards, and the accuracy of data interpretation.

## 5. Conclusions

This secondary analysis suggests that a Lithuanian OHIP-14 structure, consisting of two subscales—*psychological discomfort* and *physical discomfort*—may offer a potentially useful approach for investigating oral discomfort (OD) as a construct related to what is commonly labeled as OH-QoL and may therefore be more appropriately referred to as OD. However, the findings are preliminary and at this stage provide insufficient evidence to support the clinical use of this newly developed structure of OD regarding both *psychological* and *physical* dimensions. Further research, including validation studies with the translated and adapted OD scale, supported by these findings and with larger and more diverse sample sizes, is needed to establish its reliability, validity, and clinical utility before the scale can be recommended for use in Lithuanian clinical practice or for research to assess psychological and physical aspects of OD or to evaluate post-treatment changes.

## Figures and Tables

**Table 1:** Item–total correlations and Cronbach’s alpha if an item for deleted the 11-item OD measurement [2,9] derived from the Lithuanian OHIP-14 version [7] (*N* = 54).

Oral Discomfort	Item–Total Correlation	Cronbach’s Alpha If Item Deleted
***Psychological discomfort***		
1: Have you been self-conscious because of your teeth, mouth, or dentures?		
(Q5: Ar Jums teko nerimauti dėl dantų problemų?)	0.727	0.928
2: Have you been a bit irritable with other people because of problems with your teeth, mouth, or dentures?		
(Q11: Ar Jums teko būti suirzus dėl problemų su Jūsų dantimis, burna ar protezais?)	0.874	0.909
3: Have you been a bit embarrassed because of problems with your teeth, mouth, or dentures?		
(Q10: Ar Jums teko būti sutrikus dėl problemų su Jūsų dantimis, burna ar protezais?)	0.800	0.918
4: Have you felt tense because of problems with your teeth, mouth or dentures?		
(Q6: Ar jautėte įtampą dėl problemų su Jūsų dantimis, burna ar protezais?)	0.826	0.915
5: Have you felt that life in general was less satisfying because of problems with your teeth, mouth, or dentures?		
(Q13: Ar apskritai jautėte mažesnį pasitenkinimą gyvenimu dėl problemų su Jūsų dantimis, burna ar protezais?)	0.778	0.920
6: Have you found it difficult to relax because of problems with your teeth, mouth, or dentures?		
(Q9: Ar Jums buvo sunku atsipalaiduoti dėl problemų su Jūsų dantimis, burna ar protezais?)	0.803	0.917
***Physical discomfort***		
7: Have you had painful aching in your mouth?		
(Q3: Ar jautėte skausmus burnoje?)	0.306	0.791
8: Have you had difficulty doing your usual jobs because of problems with your teeth, mouth, or dentures?		
(Q12: Ar turėjote sunkumų atliekant įprastus darbus dėl problemų, susijusių su Jūsų dantims, burna ar protezais?)	0.425	0.756
9: Have you found it uncomfortable to eat any foods because of problems with your teeth, mouth, or dentures?		
(Q4: Ar jautėte nepatogumą valgant kokį nors maistą dėl problemų su Jūsų dantimis, burna ar protezais?)	0.610	0.692
10: Have you had to interrupt meals because of problems with your teeth, mouth, or dentures?		
(Q8: Ar turėjote pertraukti valgymą dėl problemų, susijusių su Jūsų dantims, burna ar protezais?)	0.658	0.671
11: Has your diet been unsatisfactory because of problems with your teeth, mouth, or dentures?		
(Q7: Ar Jūsų mityba buvo nepatenkinama dėl problemų, susijusių su dantims, ¿burna ar protezais?)	0.689	0.658

## Data Availability

The raw data supporting the conclusions of this article will be made available by the authors on request.

## Abbreviations

The following abbreviations are used in this manuscript:

OHIP	Oral Health Impact Profile
OD	Oral Discomfort
OH-QoL	Oral Health-related Quality of Life
LUHS	Lithuanian University of Health Science
EFA	Exploratory Factor Analysis
